# Supplementary material for: Learning curve and surgical outcome of robotic assisted colorectal surgery with ERAS program
Source: Sci Rep. 2022 Nov 29;12:20566. doi: 10.1038/s41598-022-24665-w (PMC9709162; doi:10.1038/s41598-022-24665-w)
Supplement: Supplementary file 1 — Supplementary Information 1. [file 41598_2022_24665_MOESM1_ESM.zip › LSC Raw data-Ñ[▒K/093/o_ñw╝╨░O▒Kñσ.pdf]

正式摘要(電子簽章：000000199877738)

請選擇...reporter

臺中榮民總醫院

Operation Note

[redacted]

- 手術日期：2020/09/24
- 手術醫師：VS 蔣鋒帆，CR 黃伲霖，CR 楊曙榮，CR 林昌霖，R 楊捷儒
- Anesthesia : GE
- Position : Supine
- Aseptic agents : Betadine Alcohol

Diagnosis

- Pre-operative diagnosis : Malignant tumor of T-colon, GBS
- Post-operative diagnosis : Ditto.

Surgical Indication

- For oncological resection

Operation

- Laparoscopic right colectomy and anastomosis

Finding

- Operative findings:
  1. A 3x3cm Borrmann type III ulcerative tumor at hepatic flexture. No enlarged lymph nodes at mesocolon and at ileocecal vessel root. cT3N0M0.
  2. No other gross tumor nor polyp was found.
  3. There was no visible liver metastasis, no visible peritoneal seeding.
  4. Gall bladder was removed by GS Dr.

  Operative methods:
  1. Laparoscopic right hemicolectomy with side-to-side anastomosis,(GIA 80/3.8*2).
  2. Vessel ligation : ileo-colic and right colic
  3. Cut margin: (A-colon) proximal 15cm, distal 5cm; .
  4. No penrose placed .

Procedure

- General endotracheal anesthesia was induced and then the patient was placed in the modified Trendelenburg lithotomy position with legs in adducted position. The abdomen was prepped and draped in standard sterile fashion. A small vertical incision was made above the umbilicus and a 12mm trochar was inserted into the peritoneal cavity by open method. Pneumoperitoneum was established with CO2 inflation. Laparoscope was inserted and then three 12mm trochars were inserted over RLQ, LUQ, and LLQ of abdomen. One 5mm trochar was inserted over RUQ of abdomen. The liver, omentum and peritoneum were inspected for evidence of metastatic disease. No metastatic disease was noted. The small bowel was inspected and retracted to downward by grasper. The peritoneum overlying the mesentery was then scored with electrocautery and the ileocolic artery was identified, ligated with Ligasure and endoclips. The remaining vessels to the cecum were also divided and ligated. Using electrocautery, omentum was freed from the transverse colon and lesser sac was entered. The colon was freed from its peritoneal attachments along the line of Toldt from the cecum to the hepatic flexure. Cecum, ascending colon and hepatic flexure were mobilized. The anastomosis was constructed using GIA 80/3.8mm, with Lambert suture re-inforcement. The fascia of midline wound was closed with interrupted sutures of No.1 Vicryl. The fascia of the 12 mm trochar was closed with 1-0 Vickryl and skin was closed with 3-0 Nylon. The patient tolerated the procedure well and was brought to the POR in stable condition.

- Estimated blood loss: minimum
- 器械數量: 正確
- 紗布數量: 正確

Image

- PAINT
  - colon & rectum

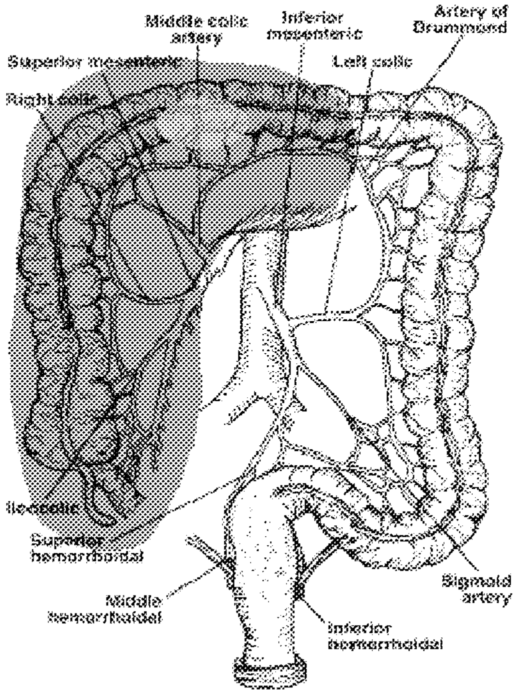

- 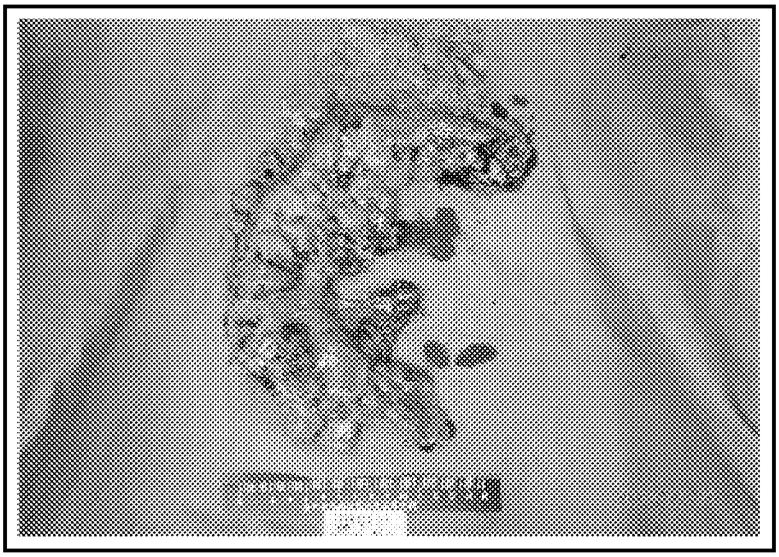

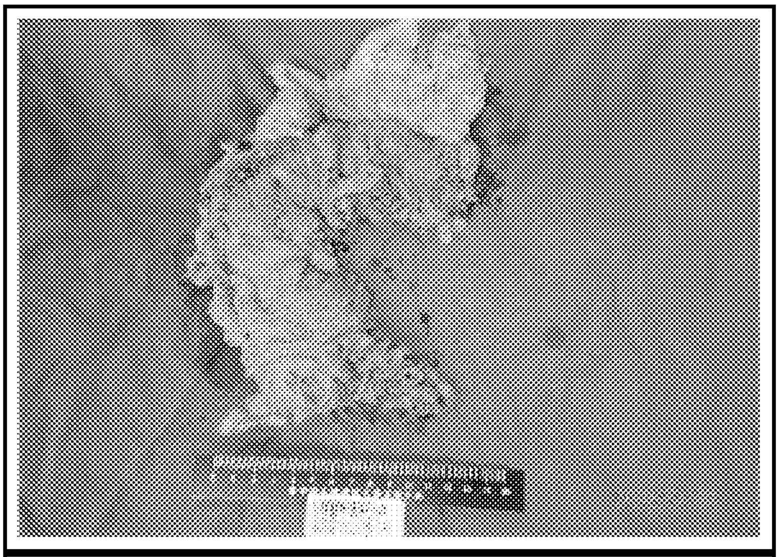

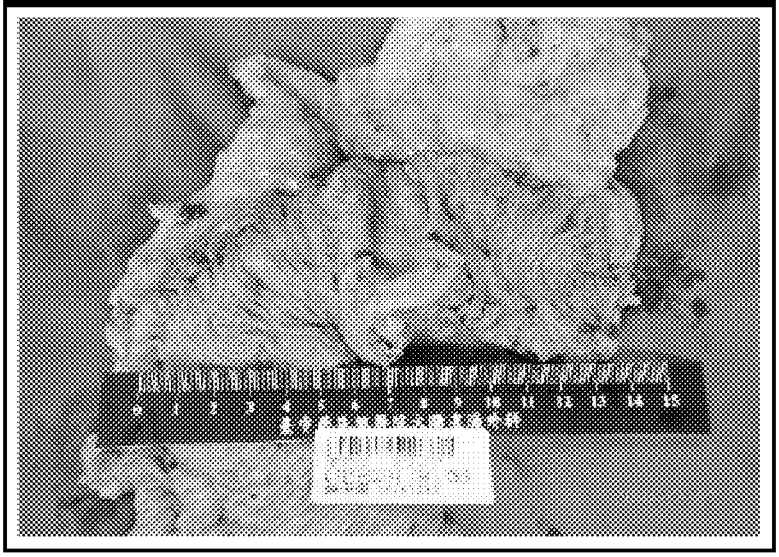

編輯主任 2062F蔣鋒帆、主治 2286H黃俋霖

正式摘要**(電子簽章：000000199877738)**

請選擇...reporter

doctorRank:B
